# Identification of potential synthetic lethal genes to p53 using a computational biology approach

**DOI:** 10.1186/1755-8794-6-30

**Published:** 2013-09-11

**Authors:** Xiaosheng Wang, Richard Simon

**Affiliations:** 1 Department of Genetics, Cell Biology and Anatomy, University of Nebraska Medical Center, Omaha, NE, USA; 2 National Cancer Institute, National Institutes of Health, Biometric Research Branch, Rockville, MD, USA

**Keywords:** Cancer, p53 mutations, Synthetic lethal genes, Gene expression profiles, Computational biology

## Abstract

**Background:**

Identification of genes that are synthetic lethal to p53 is an important strategy for anticancer therapy as p53 mutations have been reported to occur in more than half of all human cancer cases. Although genome-wide RNAi screening is an effective approach to finding synthetic lethal genes, it is costly and labor-intensive.

**Methods:**

To illustrate this approach, we identified potentially druggable genes synthetically lethal for p53 using three microarray datasets for gene expression profiles of the NCI-60 cancer cell lines, one next-generation sequencing (RNA-Seq) dataset from the Cancer Genome Atlas (TCGA) project, and one gene expression data from the Cancer Cell Line Encyclopedia (CCLE) project. We selected the genes which encoded kinases and had significantly higher expression in the tumors with functional p53 mutations (somatic mutations) than in the tumors without functional p53 mutations as the candidates of druggable synthetic lethal genes for p53. We identified important regulatory networks and functional categories pertinent to these genes, and performed an extensive survey of literature to find experimental evidence that support the synthetic lethality relationships between the genes identified and p53. We also examined the drug sensitivity difference between NCI-60 cell lines with functional p53 mutations and NCI-60 cell lines without functional p53 mutations for the compounds that target the kinases encoded by the genes identified.

**Results:**

Our results indicated that some of the candidate genes we identified had been experimentally verified to be synthetic lethal for p53 and promising targets for anticancer therapy while some other genes were putative targets for development of cancer therapeutic agents.

**Conclusions:**

Our study indicated that pre-screening of potential synthetic lethal genes using gene expression profiles is a promising approach for improving the efficiency of synthetic lethal RNAi screening.

## Background

Because mutations in the p53 tumor suppressor gene have been reported to occur in more than half of all human cancer cases [1], anticancer drugs targeting p53 mutant tumor cells are potentially efficacious for a large number of patients with cancer. Whereas p53 mutations are not directly druggable, its synthetic lethal partners may include direct drug targets. Two genes are synthetic lethal if dis-regulation of either alone doesn’t result in cell death but dis-regulation of both leads to death of cells [2]. Thus, abrogation of a gene that is synthetic lethal to p53 should selectively kill p53-mutant cancer cells and spare normal cells without p53 mutations. Based on this conceptual framework, protein products of the genes that are synthetic lethal to p53 mutations provide promising drug targets. Therefore, identification of genes synthetic lethal to p53 mutations is a viable strategy for anticancer drug development. The standard method for identifying synthetic lethal genes is based on genome-wide or kinome-wide RNAi screening which has been extensively utilized to identify sensitizing targets to chemotherapeutic agents [3]. However, large-scale synthetic lethal RNAi screening strategy is costly and labor-intensive. It is often restricted to the examination of a single exposure time and a single dose with few replicates, which may increase the false negative rates of the assay [4]. An alternative proposal for identifying synthetic lethal genes compares the gene expression profiles of isogenically paired cell lines (tumor suppressor genes mutant versus wild type), and identifies differentially expressed genes between the two cell lines. Then a gene-silencing by siRNA is performed on the differentially expressed genes to examine their synthetic lethality to the tumor suppressor gene [4]. Obviously, the gene expression profiles based method is cost-saving and potentially efficient in identification of synthetic lethal genes. Some investigators have used the method to find synthetic lethal genes [5,6].

In the present study, we identified candidate synthetic lethal genes to p53 using gene expression profiles. The kinase-encoding genes which had higher expression in the tumors with functional p53 mutations than in the tumors without functional p53 mutations (non-functional p53 mutations plus p53 wild-type) were regarded as the candidates of druggable synthetic lethal genes to p53. For purposes of the analyses here, we consider p53 nonsense (stop codon), frameshift and missense mutations as functional p53 mutations, and p53 silent mutations as non-functional p53 mutations. The silent mutations include synonymous mutations and mutations affecting noncoding DNA. Further, we identified important regulatory networks and functional categories pertinent to the candidate p53 synthetic lethal genes. We also performed an extensive examination of literature to evaluate other evidence for the putative synthetic lethality relationships between the identified genes and p53. In addition, we examined the drug sensitivity differences between NCI-60 cell lines with functional p53 mutations and NCI-60 cell lines without functional p53 mutations for the compounds that target the kinases encoded by the genes identified.

## Methods

### Identification of candidates of druggable synthetic lethal genes to p53

We first identified differentially expressed genes between the tumors with functional p53 mutations and the tumors without functional p53 mutations using the univariate F-test or t-test at a two-sided significance level of 0.05. We also performed univariate permutation tests with 10,000 permutations of the class label (functional p53 mutation or not) to measure the significance of individual genes. The proportion of the permutations that gave a t-test or F-test p value as small as obtained with the true class labels was the univariate permutation p value for that gene. We also reported the false discovery rate for each gene identified. The false discovery rate was estimated using the method of Benjami and Hochberg [7]. This procedure was implemented with the class comparison between groups of arrays tool in BRB-ArrayTools, an integrated package developed by Simon et al. for the visualization and statistical analysis of gene expression data [8]. The software can be freely downloaded from the website: http://linus.nci.nih.gov/BRB-ArrayTools.html. We selected the genes which showed higher relative expression in the tumors with functional p53 mutations and encode kinases from the differentially expressed gene list as the candidates of druggable synthetic lethal genes for p53.

### Functional annotation for the candidate genes

We inferred significant networks and biological functions associated with the candidate p53 synthetic lethal genes using Ingenuity Pathway Analysis tool (IPA, Ingenuity® Systems, http://www.ingenuity.com). IPA is a system that yields a set of networks relevant to a list of genes based on the preserved records contained in the Ingenuity Pathways Knowledge Base (IPKB).

### Comparison of drug sensitivity between two groups of cell lines

We compared drug sensitivity (GI50) between the cell lines with functional p53 mutations and the cell lines without functional p53 mutations using t-test statistics (one-sided, the hypothesis of higher sensitivity in cell lines with functional p53 mutations). GI50 is the concentration required to inhibit growth of cancer cell lines by 50%. The lower GI50 value means higher drug sensitivity. We obtained the normalized negative log(GI50) values (z-score) for more than twenty thousands of compounds from the CellMiner database [9,10].

### Materials

We selected five gene expression datasets to perform computational analysis. The five datasets include three mRNA expression datasets of NCI-60 cancer cell lines, one mRNA expression dataset of glioblastoma multiforme (GBM) from the Cancer Genome Atlas (TCGA) project, and one mRNA expression dataset of cancer cell lines from the Cancer Cell Line Encyclopedia (CCLE) project, which can be downloaded from the Developmental Therapeutics Program NCI/NIH website: http://dtp.nci.nih.gov/mtargets/download.html, the Cancer Genome Atlas website: http://tcga-data.nci.nih.gov/tcga/, and the Cancer Cell Line Encyclopedia website: http://www.broadinstitute.org/ccle/data/browseData?conversationPropagation=begin, respectively. We obtained the p53 mutation data of NCI-60 cancer cell lines from the Wellcome Trust Sanger Institute Cancer Genome Project website: http://www.sanger.ac.uk/genetics/CGP/NCI60/. Table 1 is a summary of the five gene expression datasets. The p53 mutation information for the NCI-60 cancer cell lines, the TCGA tumor samples and the CCLE cancer cell lines is provided in the supplementary Additional file 1: Table S1.

**Table 1:** Summary of the five gene expression datasets

**Dataset**	**# Genes**	**Class **^**g**^	**# Samples **^**h**^
NCI-60 Dataset 1 ^a^	1092 ^f^	c1 / c2	60 (41/19)
NCI-60 Dataset 2 ^b^	2266 ^f^	c1 / c2	60 (41/19)
NCI-60 Dataset 3 ^c^	12625	c1 / c2	59 (40/19)
TCGA Dataset ^d^	11861	c1 / c2	136 (47/89)
CCLE Dataset ^e^	18988	c1 / c2	1036 (541/495)

^a^ Affymetrix HUM6000 array data from Millenium Pharmaceuticals.

^b^ cDNA array data from the Weinstein (NCI) and Brown & Botstein (Stanford) groups.

^c^ Affymetrix U95A data from Novartis.

^d^ Gene expression data (RNA-Seq) for glioblastoma multiforme (GBM).

^e^ mRNA expression data for cancer cell lines (Affymetrix U133+2 arrays).

^f^ Number of genes filtered by excluding the genes with missing expression in at least one sample.

^g^ c1: functional p53 mutation; c2: non-functional p53 mutation or p53 wild-type.

^h^ The sample size of each class is given in parenthesis.

## Results

### Candidates of druggable synthetic lethal genes to p53

We identified 8, 2, 21, 50 and 36 gene candidates for synthetic lethality to p53 for the NCI-60 Dataset 1, 2, 3, TCGA Dataset, and CCLE Dataset respectively. Among them, PLK1 was identified in four different datasets, and CDK16, RYK, MTOR, STK17B, PLK4, MAST2, MAP3K4, MARK2, CDK1, NEK2, PRKCSH, AURKA, BUB1, CDC7, SRPK1, TTK and VRK1 were identified in two different datasets. Table 2 lists these genes accompanying with references related to them. The complete gene list (98 genes), and the genes identified in respective datasets are presented in the supplementary Additional file 2: Table S2. Experimental evidences have shown that many of our identified genes have interactions with p53. For example, in Table 2, a very interesting gene identified as synthetic lethal to p53 is PLK1 which has been found to have higher expression level in tumors with functional p53 mutations than in tumors without functional p53 mutations in four of the five datasets. Experimental evidence has shown that proto-oncogene PLK1 is involved in p53 related pathways in that PLK1 inhibits transactivation and pro-apoptotic functions of p53 function by physical interaction and phosphorylation [11]. Interestingly, it has been found that PLK1 expression is upregulated in the case of Retinoblastoma tumor suppressor (RB) inactivation, suggesting that PLK1 may be also a target of the RB pathway [12].

**Table 2:** The candidate genes with synthetic lethality to p53 identified in at least two different datasets

**Symbol**	**Name**	**Function**	**Reference**
PLK1	polo-like kinase 1	cell cycle regulation	[13-19]
CDK16	cyclin-dependent kinase 16	cell cycle regulation	[20]
RYK	receptor-like tyrosine kinase	cellular growth and differentiation Regulation	[21]
MTOR	mechanistic target of rapamycin (serine/threonine kinase)	cellular metabolism, growth, and proliferation Regulation	[22-24]
STK17B	serine/threonine kinase 17b	positive regulation of apoptosis	[25]
PLK4	polo-like kinase 4	cell cycle regulation	[17,26]
MAST2	microtubule associated serine/threonine kinase 2	cellular growth and differentiation Regulation	[27]
MAP3K4	mitogen-activated protein kinase kinase kinase 4	role in signal transduction cascades	[28]
MARK2	MAP/microtubule affinity-regulating kinase 2	cell polarity and microtubule dynamics regulation	[29]
CDK1	cyclin-dependent kinase 1	cell cycle regulation	[13,15-17,30,31]
NEK2	NIMA (never in mitosis gene a)-related kinase 2	cell cycle regulation	[14-16,30]
PRKCSH	protein kinase C substrate 80K-H	roles in inflammation, cell growth, signaling and death	[32]
AURKA	aurora kinase A	cell cycle regulation	[33,34]
BUB1	mitotic checkpoint serine/threonine kinase	cell cycle regulation	[35]
CDC7	cell division cycle 7 homolog (S. cerevisiae)	cell cycle regulation	[30]
SRPK1	SRSF protein kinase 1	cellular growth and differentiation Regulation	[36]
TTK	TTK protein kinase	cell cycle regulation	[14-16,37,38]
VRK1	vaccinia related kinase 1	cell cycle regulation	[39,40]

MTOR is a serine/threonine protein kinase that regulates cell growth, cell proliferation, cell motility, cell survival, protein synthesis, and transcription [23]. It has been demonstrated that activation of p53 inhibits MTOR activity and regulates its downstream targets [24], consistent with our finding that inactivation of p53 resulted in upregulation of MTOR. Experimental evidence also revealed that p53 and MTOR can collaboratively regulate cell growth, proliferation, and death [22].

PLK4 regulates centriole duplication during the cell cycle [41]. It has been shown that p53 and SAPK (stress-activated protein kinase) pathways cooperatively regulate PLK4 activity, and inactivation of both p53 and MKK4 genes result in hyperactivation of PLK4 which often causes supernumerary centrosomes as frequently found in cancer cells [26]. NEK2 encodes a serine/threonine-protein kinase that is involved in mitotic regulation. Evidence has shown that the gene is transcriptionally repressed by p53 [42]. AURKA encodes a kinase that regulates cell cycle by involved in microtubule formation and/or stabilization at the spindle pole during chromosome segregation. The interaction between p53 and AURKA has been investigated [33,34]. BUB1 encodes a kinase involved in mitotic spindle checkpoint function. Mutation or aberrant BUB1 expression is associated with chromosomal instability, aneuploidy, and human cancer [43]. It has been reported that p53 binds BUB1 and monitors BUB1 function [35]. CDC7 encodes a protein kinase that is predominantly localized in the nucleus. It has been found that a high correlation between p53 loss and increased CDC7 expression in primary breast cancers and in the cancer cell lines [44]. An experimental study has proved its synthetic lethality with p53 [45]. TTK encodes a dual specificity protein kinase with the ability to phosphorylate tyrosine, serine and threonine, and is critical for chromosome alignment at the centromere during mitosis. It has been shown that TTK interacts with p53 through mediating the p53-dependent postmitotic checkpoint by phosphorylating p53 [38]. VRK1 encodes a member of the vaccinia-related kinase family of serine/threonine protein kinases which localize to the nucleus and promote the stability of transcriptionally active p53 molecules. The gene may regulate cell proliferation by interaction with p53 [39,40].

To summarize, a large portion of the genes in Table 2 have experimental evidences of their interactions with p53. Some of the other identified genes not present in Table 2 like BRD2, have been experimentally verified to be synthetic lethal to p53 [46].

### Functional analysis of the genes identified

**Figure 1:**
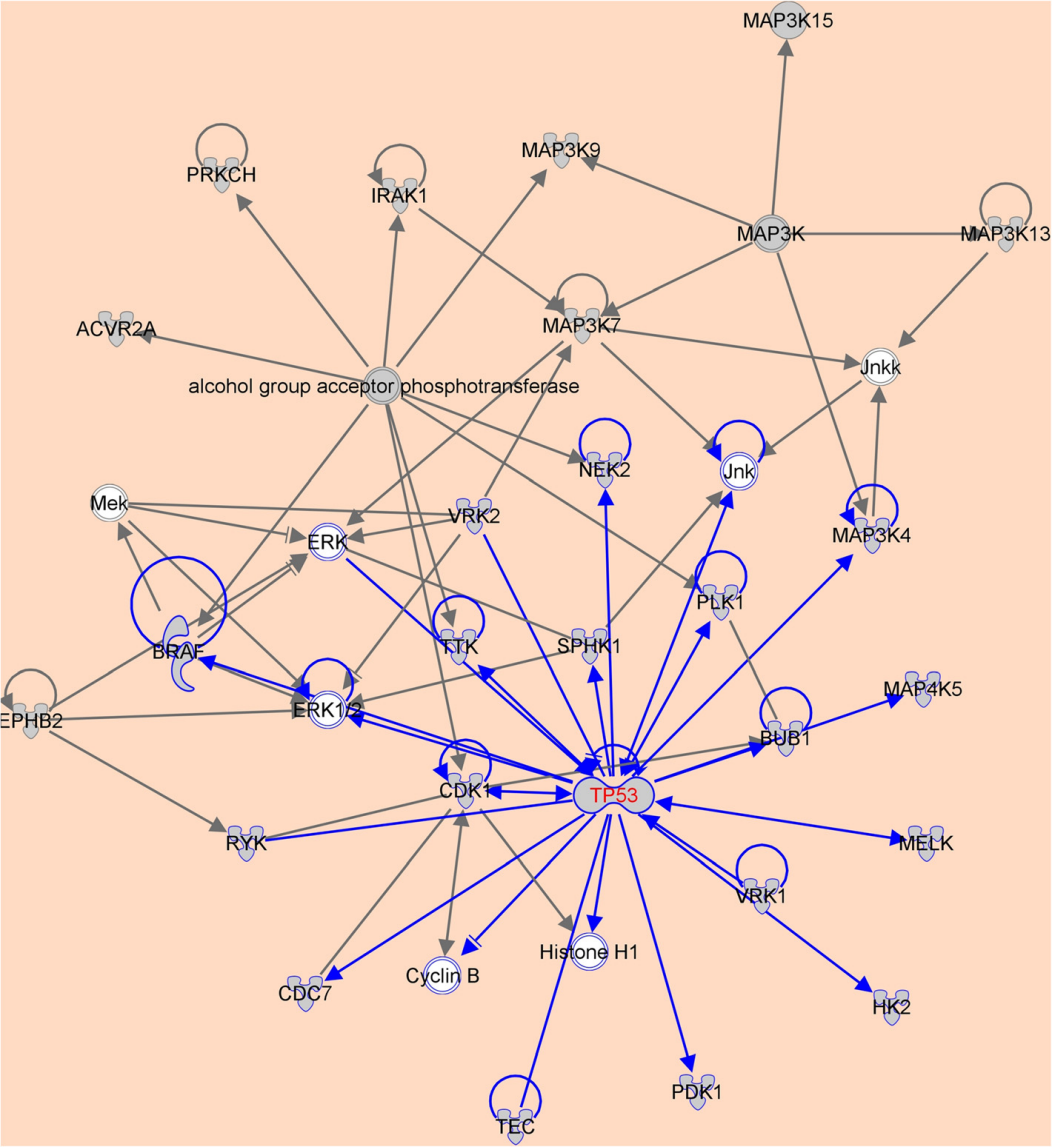
**Top scoring network associated with p53 and its 98 candidate synthetic lethal genes.** The p53-centered module is highlighted.

**Figure 2:**
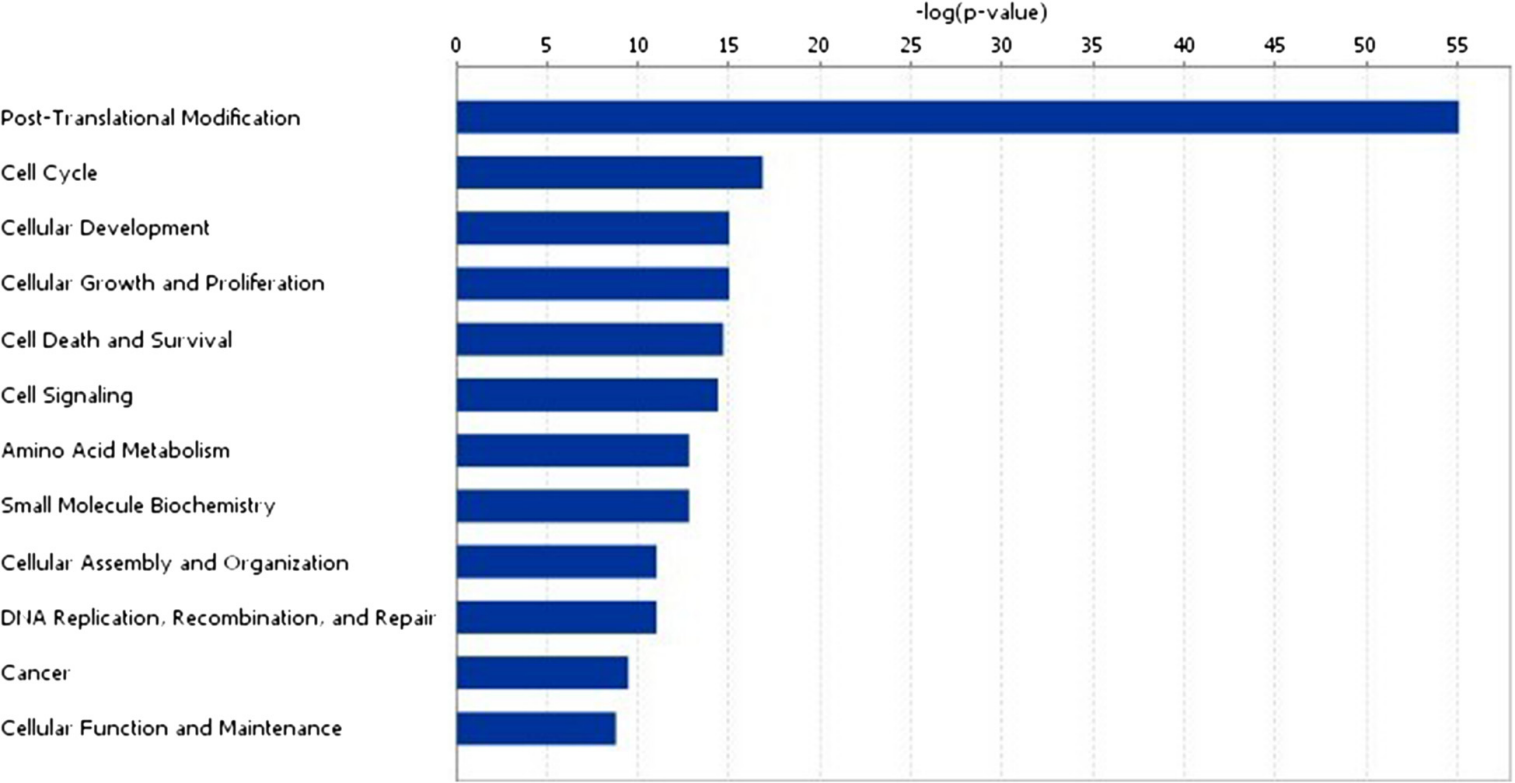
Important biological functions associated with the candidate p53 synthetic lethal genes.

Network analysis of the gene set made up of p53 and its 98 candidate synthetic lethal genes identified using IPA shows that the top network is associated with post-translational modification and cancer (Figure 1). Figure 1 shows that the network is p53-centered and many genes are its direct regulatory targets. Interestingly, half of the genes (PLK1, CDK1, NEK2, RYK, MAP3K4, BUB1, CDC7, TTK, and VRK1) presented in Table 2 are directly regulated by p53 (Figure 1), indicative of the relatedness of our identified genes to p53. Biological function analysis shows that the candidate synthetic lethal genes to p53 are mostly relevant to post-translational modification, cell cycle, cell development, cancer etc. (p-value<10^-8^, Figure 2). Table 3 presents 11 significant biological functions associated with the candidate synthetic lethal genes to p53.

**Table 3:** Important biological functions relevant to the genes identified

**Category**	**# Molecules**	**Representative genes**
Post-Translational Modification	65	RAF1,SRPK1,EPHB2,PHKG2,MARK1,MELK,MAPK13,CDK9, SRPK2,PLK4,STK17B,EIF2AK1,CHKA,RPS6KB1,MAP3K9
Cell Cycle	42	CDK1,NEK2,PIK3CA,SRPK1,CDC7,CDK11A,CDK11B,MELK, CDK9,TTK, BRAF, SRPK2,PLK4,MTOR,CLK1
Cellular Development	57	BRAF,EPHB2,CDC7,MAP3K4,MAPK13,CDK9,RIPK2,CDK16, GNE,EIF2AK1,RPS6KB1,EPHA1,AURKA,CHKA,SIK1
Cellular Growth and Proliferation	62	MTOR,EPHB2,CDC7,MELK,MAPK13,CDK9,SRPK2,PLK4, RIPK2,STK17B,EIF2AK1,GNE,NEK2,RPS6KB1,EPHA1
Cell Death and Survival	61	TTK,SRPK1,EPHB2,CDC7,MELK,MAPK13,MAP3K4,CDK9, SRPK2,PLK4,RIPK2,STK17B,GNE,EIF2AK1,MTOR
Cell Signaling	28	PLK1,SRPK1,MARK2,MARK1,MAPK13,MAP3K4,PRKCZ, IRAK1,BRAF,SRPK2,ROR2,MTOR,NEK2,CLK1,MAP3K7
Amino Acid Metabolism	15	SRPK1,EPHB2,EPHA1,ACVR1,TTK,CDK9,PHKA2,MTOR,CLK1,ABL2,PDGFRA,TLK2,CHEK2,C8orf44-SGK3/SGK3
Small Molecule Biochemistry	29	MAST2,PIK3CA,SRPK1,EPHB2,CDK16,MARK2,TTK,CDK9, PRKCZ,PHKA2,MTOR,HK2,CLK1,NTRK1,PDGFRA
Cellular Assembly and Organization	45	CHEK2,PIK3CA,SRPK1,EPHB2,MARK2,CDC7,MARK1,STK35,TTK,PRKCZ,PRKCSH,BRAF,ROR2,PLK4,MTOR
DNA Replication, Recombination, and Repair	26	ERBB3,PIK3CA,SRPK1,CDC7,TTK,PRKCZ,BRAF,PLK4,MTOR,NEK2,NTRK1,PDGFRA,SPHK1,CHEK2,PLK1
Cancer	64	TTK,SRPK1,EPHB2,CDC7,MARK1,MELK,MAP3K4,MAPK13,STK35,SCYL2,SRPK2,PLK4,STK17B,SCYL3,EIF2AK1
Cellular Function and Maintenance	43	PIK3CA,EPHB2,MARK2,MARK1,MAPK13,CDK9,TTK,STK35,PRKCZ,PRKCSH,BRAF,ROR2,PLK4,MTOR,PLK1

### Many identified genes are involved in cell cycle regulation

The repression activity of its target genes involved in the cell cycle enables p53 to control cell proliferation by inducing cell cycle arrest. Consequently, these target genes are anticipated to show higher relative expression in functional p53 mutants and that is observed in our results. Table 2 and Table 3 show that a large portion of identified genes are involved in the cell cycle regulation which include CDK1, CHEK2, TTK, BUB1B, CDC7, PLK1, PLK4, CDK11A, AURKA, VRK1, MTOR, NEK2 etc. Among them, CDK1 encodes the protein which is a member of the Ser/Thr protein kinase family, and is critical for cell cycle G1/S and G2/M phase transitions. Accumulated evidence has shown that this gene is a target of p53 transcriptional repression and had elevated expression in p53 loss/mutation status [15,16,30,31,47]. Oncogenic properties of PLK1 have been believed to be due to its role in driving cell cycle progression. In fact, PLK1 is an early trigger for G2/M transition and supports the functional maturation of the centrosome in late G2/early prophase. Its expression reaches peak during G2/M phase. CDC7 encodes a cell division cycle protein kinase that is involved in regulation of the cell cycle at the point of chromosmal DNA replication, and is specifically critical for the G1/S transition [48].

Mitosis is one of the most dramatic stages during the cell cycle. Any errors in this process often lead to aneuploidy, genomic instability, and tumorigenesis. The regulation of mitosis relies significantly on the protein phosphorylation of mitotic kinases. The important mitotic kinases include several families of kinases: CDK, POLO, AURORA and NIMA, and the mitotic checkpoint kinases [49]. Table 4 lists the identified genes that encode mitotic kinases and are classified based on the aforementioned categories. Increasing evidence suggests that p53 regulates the expression and function of many mitotic kinases and multiple mitotic kinases can also be involved in p53-mediated signaling by phosphorylation of p53, suggesting active interactions between p53 and mitotic kinases in the cell cycle regulation [50]. Our results lent a support for the argument.

**Table 4:** The identified genes encoding mitotic kinases

**Category **^i^	**Gene**
CDK Family	CDK1, CDK2, CDK9, CDK11A, CDK11B, CDK19
POLO Family	PLK1, PLK4
AURORA Family	AURKA
NIMA Family	NEK2
mitotic checkpoint	BUB1,TTK

^**i**^ Some kinases regulating mitosis may not be presented in the table such as MAP kinases.

It has been shown that the mitogen-activated protein kinase (MAPK) signaling pathways play important roles in control of the eukaryotic cell cycle, and the control of cell cycle progression by MAPK pathways is p53 dependent [51-54]. We have identified a group of MAPK pathways related genes that are potentially synthetic lethal to p53. These genes include RAF1, MAP3K13, MAP3K15, MAP3K4, MAP3K7, MAP3K9, MAP4K5, MAPK13, MAPK14, MAPKAPK5 etc.

Besides, many other genes such as CHKA, STK17B, RYK and VRK1 also play a significant role in regulation of cell cycle and proliferation.

### Some genes have been identified as potential targets of anticancer agents

Some of the identified genes such as PLK1, CDC7, MTOR, CHKA, TTK and RAF1 have been active or putative anticancer therapeutic targets. PLK1 has been recognized as a promising target for cancer therapy for its essential role in mitosis [15,18,19]. Our computational analysis has indicated that PLK1 is likely to be synthetic lethal with p53 as can been verified by two experimental studies. One study has shown that p53-deficient tumors have higher sensitivity towards the PLK1 inhibitor GSK461364A [55]. Another study has revealed that cell lines harboring p53 mutations are more sensitive to PLK1 inhibitors [56]. So far, some PLK1 inhibitors have demonstrated encouraging results in phase 1 or 2 clinical trials of cancer treatment. Table 5 lists some PLK1 inhibitors used for clinical trials. Interestingly, PLK1 has been found to have synthetic lethal interaction with KRAS [12]. Since many colon cancer [57], pancreatic cancer [58] and lung cancers [59] are associated with KRAS mutations, development of drugs targeting PLK1 kinase could be promising in treatment of these cancers.

**Table 5:** Approved or experimental compounds that target related genes

**Gene symbol**	**Drug & compound name **^**j**^	**Status**
PLK1	DB08059, DB07789, DB06963, DB06897, DB07186, BI 2536, cyclapolin 9, GW843682X, ADP, threonine, serine, pyridoxal, nocodazole, paclitaxel, phosphoserine, TAK-960	experimental
CDC7	PHA 767491 hydrochloride, BMS-863233, NMS-1116354	experimental
CDK1	flavopiridol, hymenialdisine, indirubin-3′-monoxime, olomoucine, SU9516	experimental
MTOR	rapamycin, everolimus	approved
CDK16	DB07766	experimental
PLK4	ADP, microplasmin, serine, fibrinogen, cysteine	experimental
STK17B	quercetin	experimental
TTK	DB01782	experimental
STK38	mercaptopurine	experimental
NEK2	DB07180	experimental
AURKA	Adenosine-5′-Diphosphate, phosphonothreonine, taxane, paclitaxel, serine, threonine	experimental
BUB1	cytosine, paclitaxel, nocodazole	experimental
SRPK1	gemcitabine, cisplatin, serine, arginine	experimental
PRKCSH	VU 0155069, cellobiose	experimental
PKC family (PRKCH, PRKCI, PRKCSH, PRKCZ)	bryostatin, aprinocarsen, enzastaurin, tamoxifen citrate, midostaurin, UCN-01	experimental
MARK2	glycogen	experimental
PDGFRA	imatinib, sorafenib, sunitinib, pazopanib, axitinib	approved
BRD2	PFI 1, sulfadiazine, dihydrofolate	experimental
CHEK2	DDUG	experimental
CERK	NVP 231	experimental
UCK2	DB03431**,** DB04272, DB02097, DB03403**,** DB02431, DB04005	experimental
AK2	DB01717	experimental
CHKA	choline	approved
RAF1	sorafenib	approved
CSNK2B	TMCB, TBB, ellagic acid	experimental

^j^ Gene-related drug compounds identified according to DrugBank, PharmGKB, Tocris Bioscience, HMDB, and/or Novoseek; The data were obtained from the website: http://www.genecards.org and http://www.cancer.gov/drugdictionary.

CDC7 has been suggested to be a promising target for the development of anticancer kinase inhibitors [45]. An experimental study has indicated that development of CDC7 kinase inhibitors may be efficacious in treatment of the aggressive p53-mutant breast cancer subtypes [60].

Many cancers occur due to disregulation of MTOR signaling, and therefore development of MTOR inhibitors has been an active field in cancer research [61,62]. Some MTOR inhibitors (e.g. rapamycin, everolimus) are beginning to be used in the treatment of cancer. Some others like rapalogs, ridaforolimus and BGT226 are currently in clinical development.

AURKA have been attractive targets for cancer treatment during past several years. Several ongoing clinical trials are assessing the anticancer efficacy of AURKA inhibitors [63]. We have identified several members of protein kinase C (PKC) gene family including PRKCH, PRKCI, PRKCSH, and PRKCZ. PKC isozymes are becoming attractive targets for therapeutic intervention because of their many cellular roles [64,65]. CHKA is an enzyme involved in the metabolism of phospholipids which has been found to play a role in the regulation of cell proliferation, oncogenic transformation and human carcinogenesis, and has been ascertained as a promising target for cancer therapy [66-69]. One study has demonstrated that inactivation of TTK inhibited cancer cell growth in vitro, suggesting that targeting the gene might be an effective anticancer strategy [37]. RAF1 encodes a MAP kinase kinase kinase (MAP3K), and is an excellent molecular target for anticancer therapy because of its important role in the control of gene expression involved in the cell division cycle, apoptosis, cell differentiation and cell migration [70].

Table 5 shows a partial list of clinically approved or experimentally active compounds that target some genes we have identified. For genes MTOR, PDGFRA, CHKA, and RAF1, there have been clinically approved compounds to target them, while for some other genes such as PLK1, CDC7, AURKA, STK38 and CDK1, there exist experimentally active compounds to target them (Table 5).

### In-vitro sensitivity of cell lines to compounds that target the identified p53 synthetic lethal genes

For 16 compounds with GI50 values available in Table 5, we compared their drug sensitivities between the NCI60 cell lines with functional p53 mutations and the NCI60 cell lines without functional p53 mutations. We found that there were ten compounds showing higher drug sensitivity in the cell lines with functional p53 mutations than in the cell lines without functional p53 mutations (Table 6). Few of the differences were statistically significant but the statistical power of the comparison was limited by the number of cell lines with functional p53 mutations. The compound paclitaxel has the smallest p-value, and exhibits inhibitory activity on three different kinases: PLK1, AURKA, and BUB1. There are six compounds whose means of drug sensitivity are slightly lower in the NCI60 cell lines with functional p53 mutations, but don’t show statistically significant difference (Table 6).

**Table 6:** Comparison of drug sensitivity between two groups of cell lines

**Compound**	**Mean of drug**		**p-value **^**n**^	**Targets**
	**sensitivity **^**k**^			
	**c1 **^**l**^	**c2 **^**m**^		
paclitaxel	0.1366	-0.2790	0.0323	PLK1, AURKA, BUB1
hymenialdisine	0.0590	-0.1359	0.2529	CDK1
olomoucine	0.0665	-0.1405	0.2311	CDK1
mercaptopurine	0.1012	-0.2037	0.1134	STK38
tamoxifen citrate	0.1003	-0.2121	0.0976	PRKCH, PRKCI, PRKCZ
imatinib	0.0425	-0.0358	0.3791	PDGFRA
sunitinib	0.0243	-0.0132	0.4384	PDGFRA
sorafenib	0.0801	-0.1779	0.184	PDGFRA, RAF1
DDUG	0.0860	-0.1811	0.1218	CHEK2
choline	0.0390	-0.2779	0.1188	CHKA
rapamycin	-0.1044	0.1784	0.8866	MTOR
everolimus	0.0095	0.1526	0.7188	MTOR
nocodazole	-0.0636	0.0026	0.5937	PLK1, BUB1
cisplatin	-0.1546	0.1984	0.9376	SRPK1
bryostatin	-0.0218	0.0958	0.6602	PKC family
axitinib	-0.0278	0.0589	0.6235	PDGFRA

^k^ mean of standardized negative log(GI50) values (higher means indicate higher sensitivity).

^l^ c1: The NCI60 cell lines with functional p53 mutations.

^m^ c2: The NCI60 cell lines with non-functional p53 mutations or p53 wild-type.

^n^ The one-sided t-test statistics (hypothesis of higher drug sensitivity in cell lines with functional p53 mutations).

## Discussion

A wealth of studies have established that about half of human cancer cases harbor p53 mutations, about 80% of which are missense mutations [71]. Therefore, cancer therapeutic strategies that focus on cells harboring p53 mutations are needed. Because tumor suppressor genes such as p53 are not druggable, it is rational to develop anticancer agents for druggable genes which have synthetic lethal interaction with p53. Although the genome-wide synthetic lethal RNAi screening strategy has been demonstrated to be effective in identifying potential targets for cancer therapeutic agents, pre-filtering of synthetic lethal gene candidates by the computational approach could enhance the efficiency of synthetic lethal RNAi screening. In the present study, we tried to evaluate this approach for identifying synthetic lethal p53 candidate genes using gene expression data. The results are generally promising as many of the identified genes have been experimentally verified to be synthetic lethal with or interacted with p53, and some of them have been suggested to be potential targets for anticancer therapy (see Table 2, Table 5 and Additional file 2: Table S2). More importantly, the p53 synthetic lethal genes we identified all encode protein kinases which have been targeted for the discovery of small molecule inhibitors as potential anticancer agents.

An important cluster of genes identified was involved in regulation of the cell cycle, in accordance with the pivotal role of p53 in cell cycle checkpoints [46,72,73]. Because p53 is a key regulator of G1/S checkpoints, and can promote cell cycle arrest or apoptosis in response to DNA damage, cancer cells with p53 mutations often have defects in the G1/S checkpoint while keep normal function in the G2/M checkpoint. As a result, abrogation of the G2/M checkpoint would be effective in promoting cell cycle arrest or apoptosis of p53-mutant cancer cells in the G2/M checkpoint which have escaped the fate of cell cycle arrest or apoptosis in the G1/M checkpoint. Therefore, the inhibition of G2/M checkpoint related genes should sensitize p53-mutant cancer cells to anticancer therapy while sparing normal cells [74]. Actually, among the p53 synthetic lethal gene candidates we identified, many are involved in regulation of G2/M checkpoint. A particularly interesting class of genes is the centrosome-associated regulator of the G_2_/M checkpoint such as PLK1, PLK4, CDK1, AURKA, and NEK2. In fact, centrosome has been found to play an important role in G_2_/M checkpoint function in that a growing number of G_2_/M checkpoint regulators have been found in the centrosome [75].

p53 functions are ultimately mediated by activation and repression of target genes. Wild-type p53 can induce growth arrest or apoptosis in response to stress signals such as DNA damage, UV radiation, hypoxia and chemotherapeutic agents by activation of genes which promote apoptosis or growth arrest while repression of genes involved in cell cycle and proliferation [76]. The genes identified in our study mostly belong to the target genes repressed by p53. The elevated expression of these genes is largely attributable to loss of p53 repression activity directly or indirectly, whereas some genes possibly have no connectivity with the p53 repression function at all.

As many cancer therapies tend to be less effective in p53 mutant patients, the use of small molecule inhibitors that target p53 synthetic lethal genes may enhance chemotherapeutic efficacy for these patients. Among the gene list in Table 2, in addition to the genes which have been experimentally verified to be synthetic lethal with p53 by RNAi screening, other genes are worth further investigation using RNAi screening because all of them encode druggable kinases.

Generally speaking, our gene expression profiles based pre-screening of potential p53 synthetic lethal genes provides an approach to identifying candidate genes for more extensive synthetic lethal RNAi screening, and may be useful in some cases to supplement the standard method.

Here we have used a relatively loose significance level (p-value<0.05) to identify differentially expressed genes when thousands or tens of thousands genes were tested. We didn’t use more stringent cutoff mainly considering that the number of kinase-encoding genes occupy a small proportion in all genes, and a smaller cutoff may filter out most of the kinase genes in the small-sample datasets (NCI60 cell lines datasets). In fact, for the larger datasets (TCGA and CCLE), most of the identified kinase genes have very small p-values (see Additional file 2: Table S2). If we restrict the analysis of differentially expressed genes to kinase genes, the 0.05 of cutoff would be more adequate as the number of tested genes dramatically decreases. Because the FDR calculation depends on the number of genes tested, we will have many fewer tests to correct for and the FDR identified in supplementary Additional file 2: Table S2 will decrease dramatically.

It should be noted that gene expression differences between the tumors with functional p53 mutations and the tumors without functional p53 mutations do not establish synthetic lethal relationships between differentially expressed genes and p53. RNAi screen provides a more direct way of establishing synthetic lethality relationships. Moreover, gene expression differences may not be a result of altered p53 mutation status. Some indirect or unrelated effects may exist. For example, some other genes (or pathways) other than p53 may influence the expression of the genes identified. In the present study, we have only identified candidate p53 synthetic lethal genes. The reliability of the results should be validated by RNAi screening or in-vivo experiments. Direct validation by in-vivo experiments using available kinase inhibitors may in some cases be the most direct approach since synthetic lethality screening itself needs such validation because of off-target effects.

An alternative approach to treating the p53-mutant tumors is to restore p53 tumor-suppressive function [77]. However, this is a more challenging field in that it is more difficult to develop a drug that reactivates the function of an inactivated gene than to develop a drug that inhibits the function of a hyperactivated gene, although reconstitution of the p53 pathway is believed to be an exciting novel therapeutic challenge for cancer therapeutics [78-83].

## Conclusion

Pre-screening of potential synthetic lethal genes using gene expression profiles is a promising approach for improving the efficiency of synthetic lethal RNAi screening, and may supplement the standard method in some cases. However, the reliability of the method should be validated by RNAi screening or in-vivo experiments.

## Competing interests

The authors declare that they have no competing interests.

## Authors’ contributions

XW and RS conceived of the research. XW performed the research. XW drafted the manuscript. RS revised the manuscript. Both authors read and approved the final manuscript.

## Pre-publication history

The pre-publication history for this paper can be accessed here:

http://www.biomedcentral.com/1755-8794/6/30/prepub

## Supplementary Material

Additional file 1: Table S1 The p53 mutation information for the NCI-60 cancer cell lines, the TCGA tumor samples and the CCLE cancer cell lines. Click here for file

Additional file 2: Table S2 The identified candidate synthetic lethal genes to p53. Click here for file
